# Post-dural Puncture Headache Complicated by Subdural Hematoma in an Elderly Patient Following a Pencil-Point Spinal Needle Anesthesia: A Case Report

**DOI:** 10.7759/cureus.105887

**Published:** 2026-03-26

**Authors:** Abigail Finger, Benjamin Rapsas, Esha Karayi, Jenny Han, Vendhan Ramanujam

**Affiliations:** 1 Anesthesiology, Brown University, Providence, USA; 2 Medical School, Brown University, Providence, USA

**Keywords:** case report, geriatric anesthesia, post-dural puncture headache, spinal anesthesia, subdural hematoma

## Abstract

Spinal anesthesia using a pencil-point needle in older patients is a commonly performed procedure. Post-dural puncture headache (PDPH) is a complication that can happen following spinal anesthesia due to cerebrospinal fluid (CSF) leakage, but its incidence in elderly patients is rare. While PDPH presents with typical symptoms and resolves with conservative management, in older patients, it can happen along with intracranial subdural hematoma (SDH), a rare but potentially life-threatening complication due to the persistent CSF leak. We report a rare case of a 73-year-old woman who presented with a positional PDPH, not improving with conservative treatment after undergoing elective total hip arthroplasty under spinal anesthesia with a 25-gauge pencil-point needle. While her examinations were unremarkable except for the positional PDPH, imaging studies of her head revealed a 0.34 cm SDH that remained stable. Given the non-improving PDPH and the SDH, which remained stable under close monitoring, an epidural blood patch was performed, resulting in immediate symptom resolution without further recurrence of the PDPH or worsening of the SDH. This case highlights that both PDPH and SDH, a rare combination of complications, can happen following spinal anesthesia in elderly patients. Early recognition and timely intervention, including neuroimaging and epidural blood patch, are essential to prevent morbidity and mortality.

## Introduction

Spinal anesthesia is a commonly used and safe alternative to general anesthesia for total hip arthroplasty, with studies showing fewer adverse events and better outcomes compared to general anesthesia [1,2]. Complications following spinal anesthesia can range from common but minor to rare but severe ones. Post-dural puncture headache (PDPH) is one such reported complication with a prevalence as high as 25% in the general population [3]. PDPH is a postural headache that can happen following cerebrospinal fluid (CSF) leakage through the dural puncture site, and the CSF leak is hypothesized to result in intracranial hypotension, leading to downward traction on pain-sensitive meningeal structures and worsening headache symptoms with standing. The risk factors for PDPH are both patient and procedure-related, which include young adults between 18 and 40 years of age, female gender, pregnancy, lower body mass index, previous history of PDPH, low CSF pressure, larger gauge needles, cutting needles, and multiple dural punctures [4]. PDPH symptoms typically improve with conservative management; however, persistent and severe PDPH symptoms that are refractory to conservative measures and impact activities of daily life may require an epidural blood patch (EBP) for symptom management. Furthermore, persistent or atypical symptoms or a change from positional to non-positional headache can signal a more serious underlying process and should be further investigated with cranial imaging, as PDPH may sometimes happen along with severe sequela of dural puncture such as subdural hematoma (SDH), seizures, cranial nerve palsies, and meningitis [5].

Patients who receive hip replacement surgery are generally older and are at a lower risk for spinal anesthesia-related complications, especially PDPH. The incidence of PDPH is known to be significantly less in older (>40 years) individuals when compared to the younger (<40 years) population, at less than 5% and 20%, respectively [6]. This may be due to age-related changes, such as cerebral atrophy that lowers the overall CSF pressure or a decrease in dura mater elasticity that lowers the risk of dural tears and CSF leaks in older patients. As a rare occurrence when PDPH develops after spinal anesthesia in the older population, its clinical presentation can either be typical or atypical, with an underlying post-spinal SDH. Older adults are more susceptible to SDH due to their age-related brain shrinkage and weakening of the bridging veins that can tear during the downward sagging of the brain following the low intracranial pressure that accompanies the post-spinal CSF leak. To our knowledge, reports of PDPH along with SDH post-spinal anesthesia in elderly patients following the use of non-cutting pencil-point needles are extremely limited. The approach in management to such a complication is not straightforward. We aim to present this in our report.

This article has been accepted for presentation as a meeting abstract at the 2026 Annual Regional Anesthesiology and Acute Pain Medicine Meeting on April 16, 2026.

## Case presentation

This case report lacks patient-identifiable information and is exempt from institutional review board review requirements under Brown University Health policy. Patient-informed consent was obtained for submission. This manuscript adheres to the case reports (CARE) guidelines.

**Figure 1:**
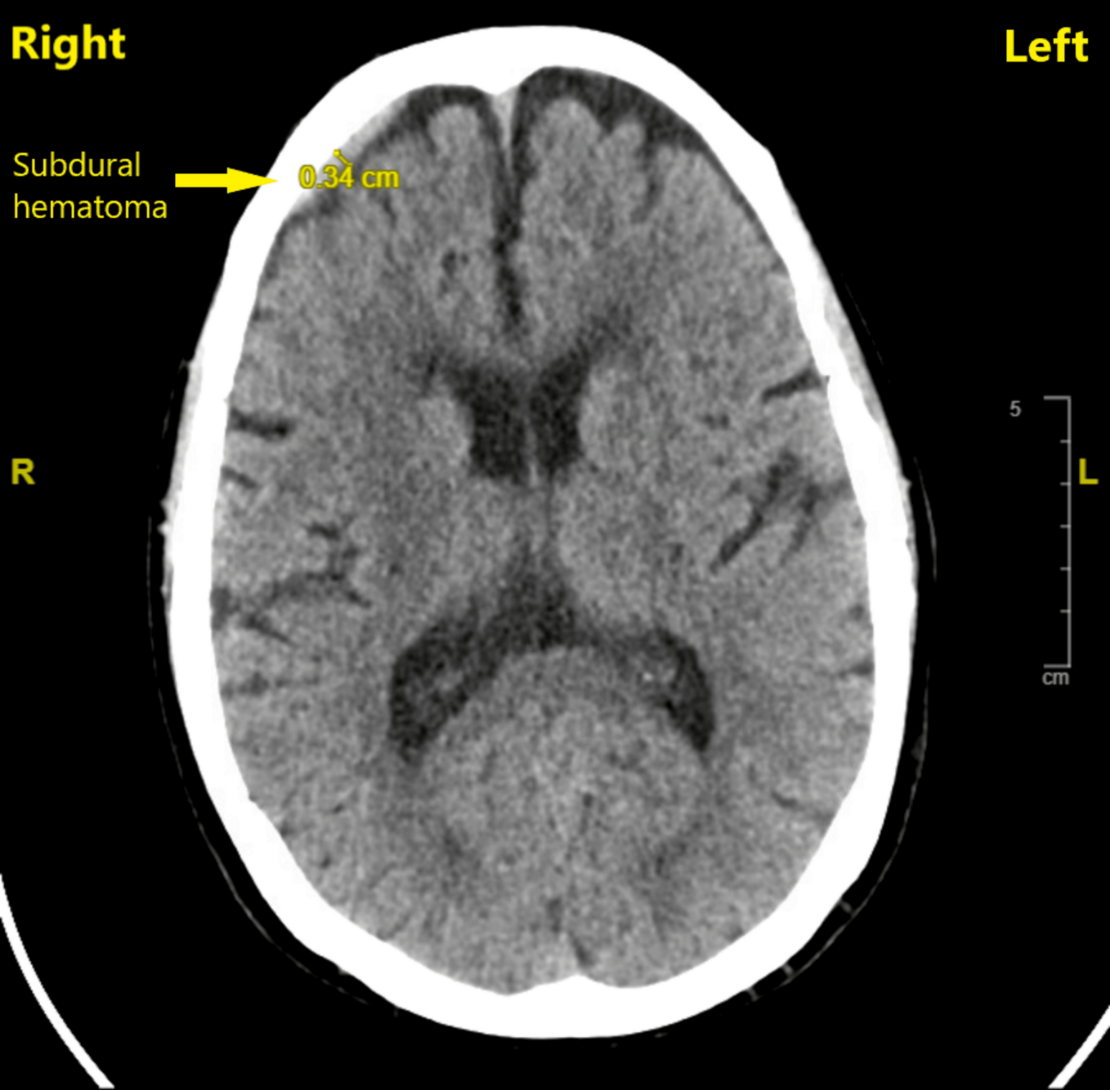
Computed tomography of the head with subdural hematoma overlying the right frontal lobe without any evidence of midline shift Computed tomography of the head performed at the initial evaluation of headache that the patient presented with five days after spinal anesthesia, revealing a subdural hematoma in the right frontal lobe without any midline shift and mass effect.

A 73-year-old female of American Society of Anesthesiologists II classification and a body mass index of 21.6 kg/m^2^, who had an elective left total hip replacement under spinal anesthesia five days ago, arrived at the emergency department due to a headache. She reported the headache in the back of her head that had started acutely after she reached home following her discharge the day after her surgery and had been lasting for the past four days. It was intermittent in nature and associated with neck tightness. She denied any associated vision or hearing changes, nausea, vomiting, fevers, chills, or any neurological deficits in her extremities. The headache was aggravated by sitting and standing, and relieved by lying down and with medications that she was taking for her post-surgical hip pain. Her past medical history included ocular myasthenia gravis with no recurrence, gastroesophageal reflux disease, and hypothyroidism. Her recent hip replacement surgery was performed under spinal anesthesia. Using sterile precautions, anatomical landmark recognition, and a midline approach between the lumbar third and fourth interspinous process spaces, a 25-gauge pencil-point needle under the guidance of an introducer was introduced intrathecally to administer 3.5 mL of 2% chloroprocaine to achieve surgical anesthesia, all in a single attempt. The patient then received intravenous propofol infusion for sedation, and her surgery was completed without any events. Her postoperative recovery was uneventful, and she was discharged home the next day. She had a known drug allergy to nitrofurantoin. Her medications included acetaminophen, oxycodone, senna, docusate, levothyroxine, and ezetimibe. On examination, her vital signs were within normal limits, and physical examination of her neurological and the rest of the systems were unremarkable. Her laboratory values were also within normal limits. A computed tomography (CT) scan that was performed for headache evaluation revealed a small, acute-appearing SDH measuring 0.34 cm in maximum thickness overlying the right frontal lobe without any evidence of midline shift (Figure 1).

**Figure 2:**
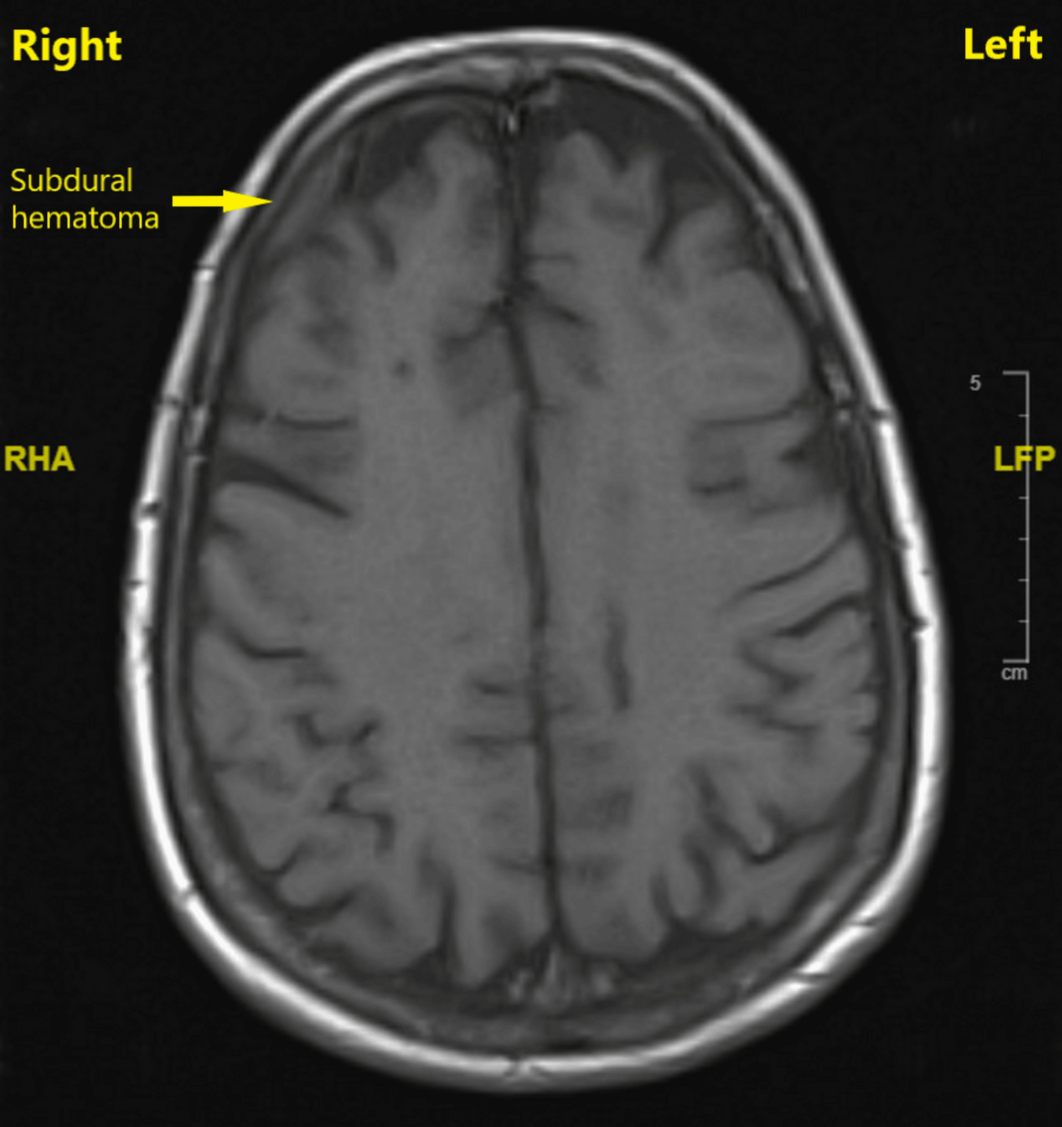
Magnetic resonance imaging of the head 6 hours later with subdural hematoma overlying the right frontal lobe without any evidence of midline shift and without any signs of intracranial hypotension Magnetic resonance imaging of the head performed 6 hours after the initial computerized tomography imaging that detected a subdural hematoma, showing that the hematoma was stable without any midline shift, mass effect, or intracranial hypotension. RHA, right head anterior; LFP, low-frequency potential.

Following a neurosurgery consultation and recommendation, after 6 hours, the CT was repeated along with a magnetic resonance imaging (MRI) of the brain, and the SDH was found to be stable without any midline shift or signs of intracranial hypotension (Figure 2).

Since the repeat scans were stable, neurosurgery recommended no further monitoring or treatment and started the patient on levetiracetam for seizure prophylaxis for one week. The diagnosis being PDPH complicated by acute SDH, the acute pain service was consulted for EBP. The decision to proceed with EBP was based on stable imaging findings, the absence of mass effect, and the persistence of orthostatic symptoms suggestive of an ongoing CSF leak, with neurosurgery recommending EBP. After discussion and obtaining consent from the patient, the EBP was performed in the same lumbar third and fourth interspinous process spaces using a 17-gauge epidural needle and 20 mL of the patient’s autologous venous blood in a sterile manner. Her headache immediately improved, and she was discharged home on the same day. Her further course of recovery during the four-week follow-up was uneventful, with complete headache resolution and no recurrence. She also did not require further imaging as per neurosurgery during her four-week follow-up period. 

## Discussion

PDPH, along with SDH occurrence following the use of a pencil-point spinal needle for spinal anesthesia in a 73-year-old elderly patient, is a rare complication, and to the best of our knowledge, has only been sparsely reported in the past [7]. The current literature on SDH as a sequela of spinal anesthesia is predominantly in obstetric patients who receive neuraxial anesthesia during childbirth, with the incidence approximately 1.5 per 100,000 deliveries overall and rising to 147 per 100,000 among those with PDPH [8,9]. The recent PDPH management guidelines from 2024 identify young age as a major risk factor for developing PDPH, and the delayed recognition or treatment of PDPH as the strongest predictor of subsequent SDH, presumably due to prolonged CSF leakage and worsening of intracranial hypotension [10]. Other known risk factors for the development of PDPH include the use of cutting needles, use of larger-size spinal needles, multiple attempts, female sex, and a previous history of PDPH. While pencil-point needles may lower the risk of PDPH, regardless of the spinal needle type and thickness, complex dural tear and delayed healing can still lead to a persistent CSF leak [11]. Puncture orifices of a diameter of 0.6 mm or more can lead to CSF leak of 240 mL/day [7]. Hence, in an elderly patient, although rare, a non-cutting pencil-point spinal needle can cause a complex dural tear, and its delayed healing can lead to PDPH development, as seen in this report.

PDPH typically presents as an orthostatic headache, worsened by sitting or standing and relieved by lying down, within five days following dural puncture, due to persistent CSF leak from the dural puncture site that can lead to intracranial hypotension. The headache is usually a frontal or occipital headache that is accompanied by neck stiffness, nausea, tinnitus, or visual disturbances. But their presentation can be atypical as well in elderly patients. In a previous report that included the elderly population, PDPH manifested as headache in only 11%, and the remaining 89% of the population presented only with symptoms other than headache, such as vomiting, diplopia, cognitive changes, changes in level of consciousness, or neurological deficits such as motor weakness or language changes [7]. The first line of management is conservative, which includes bed rest, adequate hydration, and the use of pharmacological agents such as acetaminophen, non-steroidal anti-inflammatory agents, and caffeine [12]. Other therapies that have been considered as adjuncts are abdominal binders, greater occipital nerve block, and sphenopalatine ganglion block, but their use is supported only by limited evidence [10]. If the conservative management fails after 24 to 48 hours or the PDPH is severe or debilitating, therapeutic EBP is considered [13]. EBP is an invasive intervention where the patient’s own blood, approximately 20 mL, is injected into the epidural space at a level below the existing dural leak to seal it. It has a success rate of 70% to 90%, and if it fails the first time, a second patch is done to increase the success rate to 97% [14].

As seen with this patient, during PDPH evaluation, it is important to rule out other causes of headaches, such as migraine, meningitis, cerebral venous sinus thrombosis, or SDH, especially when the headache does not resolve with the first line of conservative management. When there is a persistent CSF leak following a spinal anesthesia, the subdural veins in the brain dilate to compensate for the reduced CSF pressure, and an increasing tension starts building on them due to the caudally shifting brain. Especially in locations such as bridging veins, the wall structures of these veins are weaker, making them susceptible to a potential rupture and life-threatening SDH [8]. Elderly patients have cerebral atrophy and an increased venous vulnerability, hence placing them at risk for SDH. Studies have demonstrated this by reporting the occurrence of both acute and severe forms of SDH in older adults [15,16]. The incidence of SDH postpartum in the obstetric population following spinal anesthesia has been reported to be 1.5 per 100,000 deliveries, with some predisposing factors identified as use of anticoagulants and multiple dural punctures [8]. But their reporting in elderly patients is rare, as care in elderly patients can be systematically overlooked, leading either to nonrecognition or delayed recognition of symptoms. Cerebral atrophy and intracranial vascular abnormalities have been identified as the other predisposing factors for post-spinal anesthesia SDH development, especially in the elderly population [7]. Hence, elderly patients receiving spinal anesthesia should be considered at risk for SDH development, especially when they present with persistent, worsening, or atypical symptoms such as delayed onset of headache five days after their dural puncture window, development of focal neurologic deficits, visual disturbances, seizures, and transitioning of an orthostatic to a non-orthostatic headache.

Neuroimaging and neurosurgery consultation becomes critical in the evaluation of elderly patients presenting with headache after spinal anesthesia, where headache can happen with the background of both CSF leak and SDH that may be clinically difficult to differentiate. CT is the first-line imaging to quickly identify the bleeding. MRI is highly selective and can be used to detect the underlying hypotension and other smaller collections to guide further management. Any delay in this diagnostic work-up has been shown to lead to mortality as high as 7% and morbidity such as permanent neurological deficits as high as 11% [17,18]. Management of such a complication depends on the severity of the PDPH symptoms and the features of the SDH. The treatment is usually conservative with bedrest, hydration, and analgesics for mild PDPH symptoms, with small hematomas of <10 mm, and without brain shift or brain shift <5 mm [7]. EBP should be considered in them to treat the headache when the conservative treatment fails and when the SDH remains stable with or without evidence of intracranial hypotension in the imaging studies [19]. Severe cases with neurological deficits, large SDH, and brain shift >5 mm, though, are treated emergently with surgical evacuation of the SDH along with EBP to treat the CSF leak and prevent the hematoma recurrence [7]. The follow-up is usually until the headache is completely resolved, and the threshold should be low for reimaging the head even after an EBP when the headache returns. 

## Conclusions

This is a rare report of PDPH along with SDH in an elderly patient following spinal anesthesia, where the headache did not resolve with conservative management. Since the literature is limited on this clinical scenario, this report highlights the possibility of PDPH development in an elderly patient due to multiple factors following a spinal anesthesia, including a narrow pencil-point needle. It also highlights the development of intracranial hemorrhage in this age group, especially when there is a persistent CSF leak following a spinal anesthesia. A timely suspicion and appropriate management that includes the use of neuroimaging and EBP are key to reducing mortality and morbidity in such a scenario. 

Since this is a rare presentation, the findings and their clinical implication cannot be generalized. Future perspective includes further investigations on PDPH and SDH development in the elderly population to outline the risk factors, identify the at-risk patients, and follow them appropriately with a guideline on the use of conservative management, intracranial imaging, intervention with EBP, and surgical intervention. This will help clinical practices to continue the use of neuraxial interventions such as spinal, epidural, and diagnostic lumbar punctures in older patients safely.
